# Maternal and Neonatal Complications in Patients With Diminished Ovarian Reserve in *In-Vitro* Fertilization/Intracytoplasmic Sperm Injection Cycles

**DOI:** 10.3389/fendo.2021.648287

**Published:** 2021-04-29

**Authors:** Shuang Han, Yiwei Zhai, Qingqing Guo, Yiming Qin, Peihao Liu

**Affiliations:** ^1^ Center for Reproductive Medicine, Cheeloo College of Medicine, Shandong University, Jinan, China; ^2^ Key Laboratory of Reproductive Endocrinology of Ministry of Education, Shandong University, Jinan, China; ^3^ Shandong Key Laboratory of Reproductive Medicine, Jinan, China; ^4^ Shandong Provincial Clinical Research Center for Reproductive Health, Jinan, China; ^5^ National Research Center for Assisted Reproductive Technology and Reproductive Genetics, Shandong University, Jinan, China; ^6^ School of Basic Medical Science, Shandong First Medical University, Jinan, China

**Keywords:** diminished ovarian reserve, maternal and neonatal complications, *in-vitro* fertilization, hypertensive disorders of pregnancy, ovarian aging

## Abstract

**Background:**

Diminished ovarian reserve (DOR) is one of the most intractable clinical issues in human reproduction and is reported to be associated with raised risk of recurrent pregnancy loss and aneuploid blastocysts. In this study, we aimed to explore whether DOR was also associated with maternal and neonatal complications in *in-vitro* fertilization/intracytoplasmic sperm injection cycles.

**Methods:**

A retrospective cohort study including women below 40 years of age who achieved singleton live birth after fresh embryo transfer in *in-vitro* fertilization/intracytoplasmic sperm injection cycles in a single center from January 2012 to June 2019 was conducted. Participants with DOR, defined as basal follicle-stimulating hormone (FSH) ≥ 10IU/L and antimullerian hormone (AMH) < 1.2ng/ml, were enrolled as the study group. The controls were 1:2 matched by age and body mass index with FSH < 10IU/L and AMH ≥ 1.2ng/ml. Maternal and neonatal complications were compared between the DOR group and the controls.

**Results:**

A total of 579 women, 193 in the DOR group and 386 matched as controls, were included in this study. Compared to controls, the incidence of hypertensive disorders of pregnancy was significantly increased in the DOR group (5.7% *vs*. 2.1%, *P* = 0.021). DOR patients also presented slightly higher incidences of preterm birth (10.9% *vs*. 7.5%, *P* = 0.174) and low birthweight (6.2% *vs*. 5.4%, *P* = 0.704) yet without statistical significances. The incidences of gestational diabetes mellitus and placenta previa were comparable between the two groups.

**Conclusion:**

Compared to women with normal ovarian reserve, women with diminished ovarian reserve might have elevated incidence of hypertensive disorders of pregnancy. Patients with diminished ovarian reserve might need more strict antenatal care.

## Introduction

According to the American Society for Reproductive Medicine, diminished ovarian reserve (DOR) is defined as women of reproductive age having impaired ovarian reserve and/or poor ovarian response to gonadotropin stimulation (1), clinically characterized by elevated concentration of basal follicle-stimulating hormone (FSH), reduced antimullerian hormone (AMH) level as well as declined antral follicle count (AFC). Distinct from the low prevalence of premature ovarian insufficiency, the prevalence of DOR was relatively high, which increased from 19% to 26% from 2004 to 2011 according to a survey among the United States assisted reproductive technology population (2). For DOR patients, the impaired ovarian reserve always leads to fewer oocytes retrieved, less embryos or even no good-quality embryos acquired, which results in poor pregnancy outcomes. Additionally, with the irreversibility of reduced ovarian reserve, DOR becomes one of the most intractable situations in the clinical practice of assisted reproductive technology.

As reported, DOR is associated with raised risk of recurrent pregnancy loss and aneuploid blastocysts (3, 4); however, whether DOR is associated with maternal or neonatal complications remains uncertain. Ovarian aging has been correlated with abnormalities in luteal phase function (5), and recent studies demonstrated that luteal phase defect was related to altered maternal vascular health and higher risk of preeclampsia (6, 7). So, it is worth investigating whether the incidences of hypertensive disorders of pregnancy (HDP) and other perinatal complications are elevated among DOR patients.

The aim of the study was to explore the incidences for adverse maternal and neonatal outcomes, assessed as HDP, preterm birth (PTB), low birthweight (LBW), gestational diabetes mellitus (GDM) and placenta previa in women with DOR after *in-vitro* fertilization (IVF)/intracytoplasmic sperm injection (ICSI) treatment. 

## Materials and Methods

### Study Design and Participants

This was a retrospective cohort study of women who achieved singleton live birth after fresh embryo transfer in IVF/ICSI cycles in Center for Reproductive Medicine, Shandong University from January 2012 to June 2019. All participants were below 40 years old. Participants with DOR, defined as basal FSH ≥ 10IU/L measured at least twice and AMH < 1.2ng/ml, were enrolled as the study group. The controls were matched by age and body mass index (BMI) with FSH < 10IU/L and AMH ≥ 1.2ng/ml. To be specific, we stratified age by an interval of 5 years and in each age subgroup we stratified BMI by the category according to the World Health Organization criteria (8) (<18.5 kg/m^2^; ≥18.5kg/m^2^, <23 kg/m^2^; ≥23kg/m^2^, <27.5 kg/m^2^; ≥27.5kg/m^2^), then the controls were 1:2 enrolled. Data in this study were collected from the electronic medical record system in our center. Since female age makes huge impact on the adverse maternal and neonatal outcomes as well as the ovarian reserve, the participants were then divided into two subgroups by age with the cutoff value of 35 years old in order to further explore the associations between DOR and various pregnancy complications in different age groups. Patients with diabetes mellitus or preconceptional fasting glucose (PFG) ≥ 7.0 mmol/L, preconceptional hypertension, polycystic ovary syndrome (PCOS), thyroid dysfunction, hyperprolactinemia, endometriosis, chromosomal abnormalities, chemo-/radio-therapy or autoimmune disorders were excluded from the study. The study was approved by the Institutional Review Board (IRB) of the Center for Reproductive Medicine, Shandong University. All the participants enrolled in this study had signed written informed consent.

### Assessment of Maternal and Neonatal Complications

Maternal and neonatal complications analyzed included HDP, PTB, LBW, GDM and placenta previa. Hypertensive disorders were diagnosed according to the International Society for the Study of Hypertension in Pregnancy (ISSHP) criteria (9). HDP in this study included gestational hypertension and preeclampsia and excluded chronic hypertension. PTB was defined as live birth before 37 gestational age. LBW referred to birth weight of full-term delivered newborns below 2500 g. According to WHO criteria, GDM was diagnosed when met one or more of the following criteria: fasting plasma glucose ≥ 7.0 mmol/l; 2-h plasma glucose ≥ 11.1 mmol/l following a 75 g oral glucose load; random plasma glucose ≥ 11.1 mmol/l in the presence of diabetes symptoms (10). Placenta praevia was diagnosed when the placenta lies directly over the internal os by transvaginal ultrasound (11).

### Statistical Analysis

The baseline characteristics of patients were summarized using means ± standard deviations for continuous variables and frequencies and percentages for categorical variables. The between-group differences of continuous data were assessed by student’s t-test. Categorical data were tested by χ² analysis, with Fisher’s exact test for expected frequencies less than five. A conditional logistic regression model was also applied to adjust the factors manifesting significant differences between the DOR and control groups including AFC, basal LH concentration, days of ovarian stimulation, total gonadotropin dose and estradiol level on human chorionic gonadotropin (hCG) trigger day. All the data were analyzed by SPSS 21.0 (SPSS Inc, Chicago, IL). *P* < 0.05 was considered statistically significant.

## Results

A total of 579 women, 193 with DOR and 386 as controls, were enrolled in this study. Demographic and ovarian stimulation characteristics were presented in **Table 1**. Due to the disease attributes, between-group significant differences were observed as expected among factors including AFC (5.70 *vs*. 11.52, *P* < 0.001), AMH (0.56 *vs*. 2.92ng/ml, *P* < 0.001), basal FSH (13.77 *vs*. 6.86IU/L, *P* < 0.001), luteinizing hormone (LH, 6.01 *vs*. 4.97IU/L, *P* < 0.001), days of stimulation (9.65 *vs*. 10.99, *P* < 0.001), total gonadotropin dose (2367.68 *vs*. 1975.32IU, *P* < 0.001) and estradiol level on hCG trigger day (1468.70 *vs*. 2953.50pg/ml, *P* < 0.001).

**Table 1:** Demographic and ovarian stimulation characteristics of patients and controls during IVF/ICSI treatment.

Variables	DOR (n=193)	CON (n=386)	*P*-value
Age (year)	33.22 ± 3.78	32.71 ± 3.70	0.124
BMI (kg/m^2^)	22.83 ± 3.44	23.19 ± 3.87	0.283
Antral follicle count in both ovaries	5.70 ± 3.77	11.52 ± 4.53	<0.001*
AMH (ng/ml)	0.56 ± 0.32	2.92 ± 1.30	<0.001*
TSH (mIU/L)	2.30 ± 0.91	2.15 ± 0.85	0.059
PRL (ng/ml)	15.31 ± 6.59	16.27 ± 9.69	0.226
Baseline sex hormone			
FSH (IU/L)	13.77 ± 4.48	6.86 ± 1.44	<0.001*
LH (IU/L)	6.01 ± 4.01	4.97 ± 2.33	<0.001*
Estrodiol (pg/ml)	41.83 ± 44.18	38.30 ± 19.70	0.313
Progesterone (ng/ml)	0.59 ± 0.97	0.72 ± 2.29	0.542
Total testosterone (nmol/L)	20.33 ± 12.21	22.20 ± 10.12	0.058
Indication for IVF			
Tubal factors	117/193 (60.6%)	240/386 (62.2%)	0.717
Male factors	32/193 (16.6%)	67/386 (17.4%)	0.815
Combined factors	18/193 (9.3%)	28/386 (7.3%)	0.385
Others	26/193 (13.5%)	51/386 (13.2%)	0.931
Blood pressure			
Systolic pressure (mmHg)	117.54 ± 13.27	118.51 ± 11.07	0.353
Diastolic pressure (mmHg)	70.21 ± 9.71	71.48 ± 8.16	0.099
Preconceptional fasting glucose (mmol/L)	5.23 ± 0.55	5.43 ± 2.33	0.377
Days of ovarian stimulation	9.65 ± 3.58	10.99 ± 2.35	<0.001*
Total gonadotropin dose (IU)	2367.68 ± 1310.03	1975.32 ± 851.84	<0.001*
Estradiol level on hCG trigger day (pg/ml)	1468.70 ± 1075.11	2953.50 ± 1585.56	<0.001*
Progesterone level on hCG trigger day (ng/ml)	0.81 ± 1.03	0.95 ± 1.73	0.346

AMH, antimullerian hormone; BMI, body mass index; PRL, prolactin; FSH, follicle-stimulating hormone; LH, luteinizing hormone; IVF, in-vitro fertilization; ICSI, intracytoplasmic sperm injection; hCG, human chorionic gonadotropin. *indicates statistical significances of P < 0.05.

**Figure 1:**
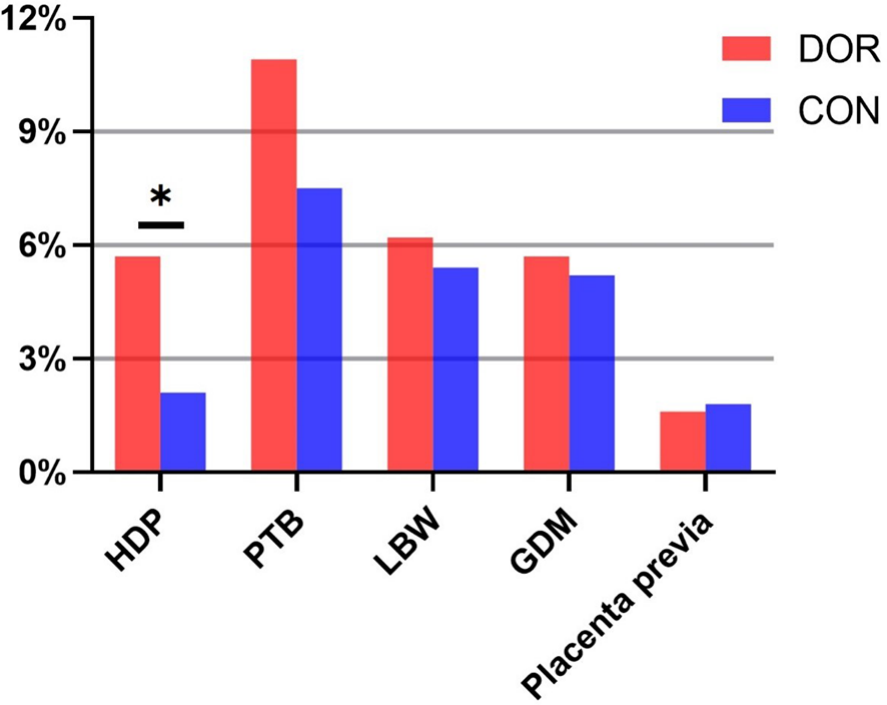
Gestational complications in DOR patients and controls. HDP, hypertensive disorders of pregnancy; PTB, preterm birth; LBW, low birthweight; GDM, gestational diabetes mellitus. *indicates statistical significance of P < 0.05.

Maternal and neonatal complications were listed in **Table 2** and presented in **Figure 1**. Compared to controls, the incidence of HDP was significantly elevated in the DOR group (5.7% *vs*. 2.1%, *P* = 0.021). DOR patients also presented slightly increased incidences of PTB (10.9% *vs*. 7.5%, *P* = 0.174) and LBW (6.2% *vs*. 5.4%, *P* = 0.704) yet without statistical significances. The incidences of GDM and placenta previa were comparable between the two groups. After the adjustment, the DOR group still exhibited a raised incidence of HDP compared to the controls (adjusted OR 2.63, 95%CI 1.01-6.84, *P*=0.045). None of the other maternal or neonatal complications had significantly different incidences between the DOR and non-DOR groups after the adjustment.

**Table 2:** Comparison of gestational complications between women with DOR and controls.

Gestational complications	DOR (n=193)	CON (n=386)	*P*-value
Hypertensive disorders of pregnancy	11 (5.7%)	8 (2.1%)	0.021*
Preterm birth	21 (10.9%)	29 (7.5%)	0.174
Low birth weight	12 (6.2%)	21 (5.4%)	0.704
Gestational diabetes mellitus	11 (5.7%)	20 (5.2%)	0.794
Placenta previa	3 (1.6%)	7 (1.8%)	1.000

*indicates statistical significance of P < 0.05.

**Table 3** demonstrated the gestational complications when stratified by age. There were 119 DOR women and 238 controls below 35 years old, leaving 74 DOR patients and 148 age matched controls in the advanced age subgroup. Although lacking of statistical differences, in younger patients with DOR, the incidences of HDP (5.0% *vs*. 1.3%, *P* = 0.065), PTB (11.8% *vs*. 6.3%, *P* = 0.075) and LBW (6.7% *vs*. 5.0%, *P* = 0/515) were increased compared with controls. However, among women over 35 years of age, only the incidence of HDP represented a rising trend in the DOR group (6.8% *vs*. 3.3%, *P* = 0.307). The incidences of all other complications were similar between the two groups in women of advanced ages. 

**Table 3:** Gestational complications stratified by age in women with DOR and controls.

Gestational complications with women＜35 years old	DOR (n=119)	CON (n=238)	*P*-value
Hypertensive disorders of pregnancy	6 (5.0%)	3 (1.3%)	0.065
Preterm birth	14 (11.8%)	15 (6.3%)	0.075
Low birth weight	8(6.7%)	12 (5.0%)	0.515
Gestational diabetes mellitus	3 (2.5%)	7 (2.9%)	0.821
Placenta previa	1 (0.8%)	4 (1.7%)	0.524
**Gestational complications with women ≥ 35 years old**	**DOR (n=74)**	**CON (n=148)**	***P*-value**
Hypertensive disorders of pregnancy	5 (6.8%)	5 (3.3%)	0.307
Preterm birth	7 (9.5%)	14 (9.5%)	1.000
Low birth weight	4 (5.4%)	9 (6.1%)	1.000
Gestational diabetes mellitus	8 (10.8%)	13 (8.8%)	0.627
Placenta previa	2 (2.7%)	3 (2.0%)	1.000

## Discussion

This study analyzed the risk of maternal and neonatal complications, including HDP, PTB, LBW, GDM and placenta previa in women with DOR after IVF/ICSI treatment. The results suggested that women with impaired ovarian function had higher incidence of HDP. And the incidences of PTB and LBW also had an elevated trend. Our study indicates that patients with DOR are supposed to be under more careful antenatal examinations for any signs of pregnancy complications.

With decreased oocyte quantity, DOR is a common status of ovarian aging. According to the committee opinion of the American Society for Reproductive Medicine, ovarian aging is associated with abnormalities of luteal phase function (5). The progesterone and estradiol metabolites production during luteal phase was decreased greatly among advanced aged women (12, 13). It has been reported that vascular problems and hypertensive disorders of pregnancy was related with luteal phase dysfunction and lacking of relaxin (6, 7).

Similar findings were reported that hormone replace therapy cycles with no corpus luteum in frozen embryo transfer had increased incidence of hypertensive disorders of pregnancy (7, 14, 15). And von Versen-Hoynck et al (6, 7) revealed that relaxin concentration in the hormone replace therapy cycles with no corpus luteum was significantly lower than that in the natural cycles, and maternal vascular health and aortic compliance was impaired remarkably in the hormone replace therapy group as well. In our study, patients with DOR, who were supposed to have poor luteal function producing less relaxin, therefore presented with elevated incidence of HDP.

Relaxin is a potent vasodilator produced only by corpus luteum throughout gestation (16). Relaxin has crucial impacts on the vascular health during pregnancy. First, lacking of or low level of relaxin in the DOR patients may results in impaired decidualization, which is the key pathological process of preeclampsia. Second, dramatic remodeling of the uterine artery wall occurs during pregnancy and relaxin is the leading candidate molecule inducing compositional and geometric remodeling of arteries (17, 18). The arterial remodeling fuels the changes of arterial passive mechanical properties which benefits the arterial compliance. Third, relaxin also has vasodilatory effect acting through increased expression of inducible nitric oxide generation (19, 20).

Another explanation for the elevated HDP incidence in women with impaired ovarian reserve could be ascribed to the deteriorative status of oxidative stress. Two cross-sectional studies indicated that serum levels of inducible nitric oxide synthase, total oxidant status, and oxidative stress index in patients with premature ovarian insufficiency were elevated significantly (21, 22). Oxidative stress could decrease the biodisponibility of nitric oxide and prostacyclin and result in increased vasoconstriction and reduced endothelium-dependent vasodilation (23). Excessive oxidative stress is proved to be associated with severe hypertension and target organ damage (24) in human and mice.

Our study also provided the clinicians with more informative clues for antenatal managements of patients with impaired ovarian reserve. HDP is one of the major causes of maternal and neonatal morbidity and mortality (25). Thus, it is crucial to identify the risk factors for HDP and take preventative measures. Since DOR is a risk factor for HDP, obstetricians need to be vigilant on patients’ ovarian reserve evaluation and pay special attention to their blood pressure for patients with DOR. For example, frequent home blood pressure monitoring will be helpful, and weight management during pregnancy is recommended as obese is a known risk factor for preeclampsia. Low-dose aspirin for prevention of preeclampsia may also be considered for DOR patients, especially for those who have other risk factors for eclampsia.

Previous studies always focused on the pregnancy outcomes of DOR patients except for one study (26) analyzing the perinatal outcomes as well; however, they only included DOR patients under 35 years old. Calhoun et al’ s study (27) was also a retrospective study focusing on the association between FSH and obstetric outcomes including PTB and LBW among women underwent IVF. With a large cohort, their study categorized the participants into three groups (defined by the serum FSH concentration of 1–5, 6–8 and ≥ 9 mIU/ml) and revealed that the incidences of PTB and LBW were not significantly different among the groups. In our study we strictly defined the women with DOR as basal FSH ≥ 10IU/L measured at least twice as well as AMH < 1.2ng/ml, which meant that we homogenized the patients with impaired ovarian reserve and aimed to figure out the characteristics of this specific population. Both two studies failed to reveal a significant association between ovarian reserve and the incidences of PTB and LBW, which might indicate that poor ovarian reserve did not affect PTB or LBW even taken AMH into consideration or lift the FSH cut-off values. The major limitation of our study was its retrospective design. The levels of relaxin and estrogen concentration during pregnancy were lacking. Information of other potential confounders including second-hand smoking exposure, income levels, nutritional status, physical activity, and sleep during pregnancy could not be collected as well. Prospective studies with larger size of patients are needed in the future.

## Conclusion

In conclusion, diminished ovarian reserve might be a potential risk factor for hypertensive disorders of pregnancy. Although it still needs further exploration with larger sample size to confirm this conclusion, patients with DOR might be benefit from more careful pregnancy surveillance.

## Data Availability Statement

The raw data supporting the conclusions of this article will be made available by the authors, without undue reservation.

## Ethics Statement

The studies involving human participants were reviewed and approved by the Institutional Review Board (IRB) of the Center for Reproductive Medicine, Shandong University. The patients/participants provided their written informed consent to participate in this study. 

## Author Contributions

In this research, PL and SH contributed to the conception and design. YZ, SH and QG were involved in data collection and analysis. PL, YQ and SH drafted and modified the manuscript. All authors contributed to the article and approved the submitted version. 

## Funding

This study was funded by Natural Science Foundation of Shandong Province (ZR2019PH009), Shanghai Commission of Science and Technology (no. 19410760300) and Taishan Scholars Program for Young Experts of Shandong Province (tsqn20161069).

## Conflict of Interest

The authors declare that the research was conducted in the absence of any commercial or financial relationships that could be construed as a potential conflict of interest.
